# Expressive writing interventions in patients with cancer: A scoping literature review

**DOI:** 10.1017/S1478951525100394

**Published:** 2025-07-21

**Authors:** Jowan Watson, Cynthia S. Peng, Marie Desir, Manfred Nathan Mate-Cole, Hermioni Amonoo

**Affiliations:** 1 Harvard Medical School, Boston, MA, USA; 2 Department of Psychiatry, McLean Hospital, Belmont, MA, USA; 3 Department of Psychiatry, Brigham and Women’s Hospital, Boston, MA, USA; 4 Division of General Medicine, Beth Israel Deaconess Medical Center, Boston, MA, USA; 5 Department of Psychosocial Oncology and Palliative Care, Dana-Farber Cancer Institute, Boston, MA, USA

**Keywords:** Expressive writing, expressive writing intervention, quality of life, cancer, psychosocial care

## Abstract

**Background:**

Expressive writing interventions (EWIs) are associated with important psychological and physical outcomes in patients with cancer. However, EWIs have not been widely integrated into routine psychosocial care of cancer populations. A review of the current literature on EWIs’ impact on the cancer patient experience, including qualitative analyses of patient perspectives, will increase our understanding of barriers and facilitators to adoption in clinical settings.

**Objectives:**

To bridge existing gaps in the literature by examining quantitative and qualitative studies on EWIs for patients with cancer. To present recent data examining the benefits of EWI’s for patients with cancer.To provide strategies for clinicians engaging in EWI’s for their patients.

**Methods:**

Informed by the Preferred Reporting Items for Systematic reviews and Meta-Analysis (PRISMA) guidelines, we completed a scoping review of relevant quantitative and qualitative articles published from 2015 to 2025 to assess the impact of EWIs on health-related outcomes (e.g., physical symptoms and quality of life [QOL]) as well as approaches to improve their use in patients with cancer.

**Results:**

Of the 28 studies with 3527 patients that we analyzed, 24 were quantitative and 4 were qualitative. Most studies were conducted in the USA (42.8%) or China (28.6%) and included patients with breast cancer (71.4%) or only included women (71.4%). Of the patients in the studies, 46.8% identified as White, 42.8% as Asian, 5.5% as Black, and 4.5% as Latino. Twenty-one of the quantitative studies found that EWIs were positively associated with cancer patients’ QOL and/or physical health outcomes. Of the 4 qualitative studies, themes of narrative reconstruction, cultural disclosure norms, and intervention delivery format emerged. The characteristics of EWI methods can be tailored to maximize therapeutic benefits through cultural adaptation, timing, and privacy.

**Significance of results:**

Despite promising associations between EWIs and health-related outcomes in patients with cancer, EWIs for cancer populations are heterogeneous and randomized clinical trials are limited. Larger trials that establish the efficacy of EWIs in diverse cancer populations are warranted.

## Introduction

Expressive writing interventions (EWIs) have attracted significant interest in the field of oncology for the past 2 decades (Merz et al. 2014). Expressive writing (EW) was originally conceptualized by Pennebaker and Beall (1986). The original paradigm was first tested on healthy undergraduate students who wrote for 20 min over 4 days about their deepest emotions and thoughts related to traumatic or upsetting experiences, with the theory positing that the act of disclosing traumatic events helps to organize, assimilate, or give meaning to trauma, ultimately serving a cathartic function (Pennebaker and Chung 2007). Pennebaker and Beall then proposed that inhibiting behavior is psychologically stressful and that the effort to suppress trauma can result in rumination. Instead, writing allows for the release of these traumatic emotions to reduce psychological stress and improve well-being (Pennebaker and Chung 2007).

EW has been of interest for patients with cancer due to its potential to address emotional inhibition, a prevalent issue among oncology patients. Since the early 2000s, patients who frequently experience trauma from a cancer diagnosis and treatment have received EWIs (De Moor et al. 2002; Rosenberg et al. 2002; Stanton et al. 2002; Walker et al. 1999; Zakowski et al. 2004). Emotional inhibition in these patients can lead to adverse psychological outcomes, a gap EW may fill by providing an outlet for emotional expression^11^. The mechanisms underlying EW, such as catharsis, cognitive restructuring of trauma, emotional regulation, and modulation of habituation to trauma-related emotions, are especially relevant to the experiences of patients with cancer (Chu et al. 2020; Lepore et al. 2015; Pennebaker and Beall 1986). EW also offers patients with cancer diagnosis the opportunity to safely explore and process cancer-related thoughts and feelings in a private setting, free from the possibility of receiving unsupportive feedback, thus promoting open expression and emotional release without inhibition (Chu et al. 2020). EWIs facilitate self-regulation skills, including increased confidence, stress management, and regulation of thoughts and behaviors, to restore a sense of perceived control often lost during cancer diagnosis and treatment (Chu et al. 2020; Lepore et al. 2015; Zachariae and O’Toole 2015). Through expressive flexibility, patients learn to adjust their emotional responses depending on the context, to engage or disengage from negative emotions as needed (Kupeli et al. 2019). Additionally, EWIs promote schema-building by enabling patients to reflect, process, and reframe their experiences, integrating negative events into a self-schema (Chung and Pennebaker 2011; Low et al. 2006). While writing may lead to distress, it ultimately cultivates greater self-knowledge and psychological resilience (Pennebaker and Beall 1986)

Despite several meta-analyses and systematic reviews investigating the impact of EWIs in cancer care, variations in intervention type and length, cancer type, and intervention assessment measures remain a barrier to their integration into clinical practice. Additionally, recent reviews have primarily examined quantitative assessments of EWI interventions without a synthesis on patient experiences with EWI from qualitative studies (Abu-Odah et al. 2024; Kupeli et al. 2019; Merz et al. 2014; Zachariae and O’Toole 2015). Hence, this scoping systematic review bridges existing gaps in the literature by examining quantitative and qualitative studies on EWIs for patients with cancer to inform ongoing work to tailor EWIs for this population.

## Methods

A scoping systematic review following the Preferred Reporting Items for Systematic reviews and Meta-Analysis (PRISMA) guidelines was conducted (Tricco et al. 2018). A scoping systematic review is relevant for this type of investigation because it allows for the inclusion of both qualitative and quantitative studies. Since EW is an intervention that lends itself to both empirical and narrative outcomes, the inclusion of all types of studies is paramount to encapsulate patient-reported qualitative and quantitative benefits of EWIs (Mak and Thomas 2022; Peters et al. 2020).

### Search strategy

We conducted a comprehensive literature search via MEDLINE/PubMed and Web of Science to identify relevant articles published over the last 10 years (January 1, 2015 to February 24, 2025). The literature search included Medical Subject Headings (MeSH) and related text and keyword searches focusing on terms to describe writing interventions in cancer populations. The initial search was conducted on August 2, 2021, yielding 19 studies, a second search was conducted on August 2, 2024, yielding 21 studies, and a third search was conducted on February 24, 2025, yielding 28 studies. We combined keywords related to the intervention (“expressive writing” or “therapeutic writing”) with the keywords related to the patient population (“cancer” or “oncology”). Both quantitative and qualitative studies were considered.

### Study selection

**Figure 1.**
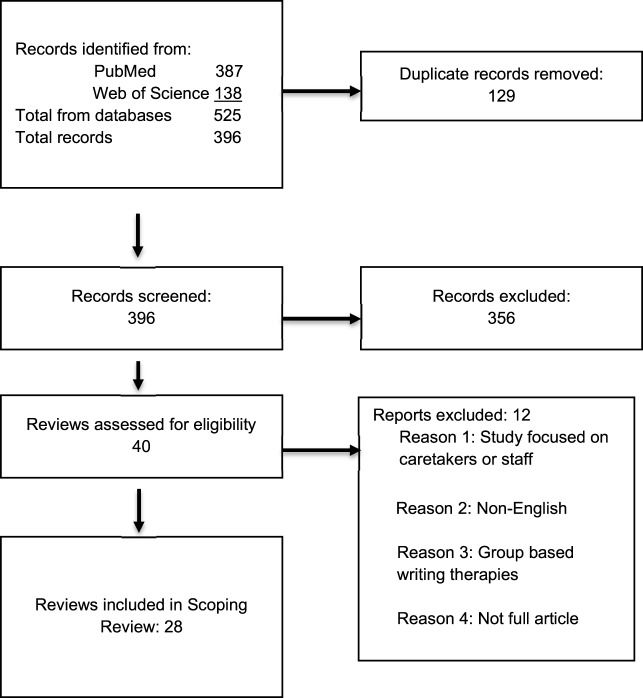
PRISMA selection of studies.

Figure 1 illustrates the selection process. Studies were independently screened by the authors J.W. and C.S.P. Disagreements were resolved by collaborative reanalysis of the studies in question.

### Inclusion and exclusion criteria

Studies included in the review had some form of EWI during the cancer care continuum. Studies included adult patients who were 18 years or older, underwent virtual and/or in-person individual interventions, experienced any malignancy, and were at any point across the cancer care continuum (including at the time of diagnosis, pre-treatment, post-treatment, in remission, and end-of-life). We excluded studies if the study subjects were caregivers or staff, involved group-based writing interventions, if there was no English version available, or if the articles were dissertations, conference proceedings, or otherwise unavailable.

## Results

### Study selection

There were 24 quantitative studies and 4 qualitative studies examined. Figure 1 illustrates the selection process. A total of 396 articles were screened for inclusion. Of these, 129 duplicate records were removed. Abstract screening of the remaining 396 yielded 40 articles for full-text assessment of eligibility, and this yielded 28 articles for inclusion in this scoping review. Table 1 provides the full details of each study and its findings. All studies included were written in English. The majority were conducted in the USA (42.8%), with the remainder of the studies conducted in China (28.6%), Australia (14.3%), Denmark (3.6%), Italy (3.6%), Indonesia (3.6%), and the Netherlands (3.6%) (Table 1).

**Table 1.** Studies (*N* = 28) reporting experimental trials of expressive writing interventions in cancer patients

Quantitative studies (*N* = 24)	Study design	Sample size (*N*)	Sample characteristics	Intervention description	Outcomes	Major findings
Lepore et al. 2015	Two-arm randomized controlled trial	*N* = 193	Adults with colorectal cancer stages 1–4 in the USA.	15-min sessions twice per week over 2 weeks. Outcomes assessed at 1-month pre and post. EW: emotional disclosure prompt (*n* = 101) Control: daily facts prompt (*n* = 92)	Extent of meaningful, personal, and emotional writing (7-point Likert scale), expression of emotion and cognitive processing scale (LIWC), social constraints (SCS), depressive symptoms (CES-D), sleep problems (Pittsburgh Sleep Quality Index), QOL indicators (QLQ-C30)	Relative to the Control group, participants in the EW group expressed more negative emotion in writing and rated their writings as more meaningful, personal, and emotionally revealing. There were no significant main effects of EW or moderating effects of sex or social constraints on outcomes (including QOL)
			Race: 85.0% White Gender: 51% male and 49% female			
Lu et al. 2017	Two-arm randomized controlled trial	*N* = 96	Adults with breast cancer stages 0–3 in the USA.	30-min sessions once per week over 3 weeks Outcomes assessed at baseline, 1, 3, and 6 months EW: self-regulation prompt (*n* = 34) Emotional disclosure prompt (*n* = 29)Control: cancer facts prompt (*n* = 33)	Health-related QOL using the 27-item Functional Assessment of Cancer Therapy Scale (FACT-G)	The cancer-fact group reported the highest level of overall quality of life (QOL) at the 6-month follow-up. The self-regulation group had higher emotional well-being compared to the emotional disclosure group
			Race: 100% Asian (Chinese) Gender: all female			
Sohl et al. 2017	Two-arm randomized controlled trial	*N* = 104	Adults with breast cancer and stage 2 lymphedema in the USA.	20-min sessions twice per week over 2 weeks. Outcomes assessed at baseline, 1, 3, and 6 months EW: emotional disclosure prompt (*n* = 51) Control: daily facts prompt (*n* = 53)	QoL related to lymphedema using 27-item Upper Limb Lymphedema scale (ULL-27), QOL using the Functional Assessment of Cancer Therapy – Breast scale (FACT-B), dispositional optimism using 10-item Life Orientation Test-Revised scale (LOT-R), Avoidance using 8-item subscale of the Impact of Event Scale (IES)	No significant intent-to-treat effects of EWI on QoL. EW was more effective for improving QoL in women who were higher on optimism, lower on avoidance, and had less time since a lymphedema diagnosis
			Race: 83.7% White Gender: all female Location: USA			
Milbury et al. 2017	Two-arm randomized controlled trial	*N* = 277	Adults with renal cell carcinoma stage 1–4 in the USA.	Four 20-min sessions over 10 days. Outcomes assessed at baseline, 1, 4, and 10 months EW: emotional disclosure prompt (*n* = 138) Control: daily facts prompt (*n* = 139)	Cancer-related symptoms using 13-item MD Anderson Symptom Inventory (MDASI), depressive symptoms using 20-item Center for Epidemiologic Studies Depression Scale (CES-D), fatigue using 9-item Brief Fatigue Inventory (BFI), sleep disturbances using 18-item Pittsburgh Sleep Quality Index (PSQI), social support using 20-item Medical Outcomes Study Social Support Survey (MOS-SSS)	EW most effective in terms of cancer-related symptoms (*p* < .05), depressive symptoms (*p* < .01), and sleep disturbances (*p* = .005) for participants with elevated depressive symptoms who received high levels of social support at baseline relative to control
			Race: 76.1% White Gender: 60% male and 40% female			
Jensen-Johansen et al. 2018	Two-arm randomized controlled trial	*N* = 507	Adult patients with breast cancer stages 1–2 in Denmark.	Three 20-min sessions over 3 weeks. Outcomes assessed at 3 and 9 months EW: emotional disclosure prompt (*n* = 253) Control: daily facts prompt (*n* = 254)	Physical symptoms using 15-item Patient Health Questionnaire Somatic Symptoms subscale (PHQ15), repressive coping style using both Marlowe–Crowne Social Desirability scale (MCSD-TMAS) and Taylor Manifest Anxiety Scale (TMAS-Bendig Short Form), alexithymia using Toronto Alexithymia Scale (TAS-20), rumination using Emotional Control Questionnaire rehearsal subscale (ECQ-R), social constraints using Social Constraints Scale (SCS-C)	Low-alexithymia women in the EW group showed larger decreases in GP telephone calls over time than both high alexithymia women and controls Women in the EW group writing about their own cancer, but not women writing about other topics, showed a larger decrease than controls
			Race: all Danish ethnicity Gender: all female			
Lu et al. 2018	Three-arm randomized controlled trial	*N* = 136	Adult patients with breast cancer stages 0–4 in the USA.	30-min sessions once per week over 3 weeks. Outcomes assessed at baseline, 1, 3, and 6 months EW: self-regulation prompt (*n* = 46) Enhanced Self-regulation prompt (*n* = 54) Control: cancer facts prompt (*n* = 36)	QOL using Functional Assessment of Cancer Therapy general scale (FACT-G)	Expressive writing is an effective intervention to improve QoL for Chinese American cancer survivors. The enhanced self-regulation condition had a large and statistically significant effect (*d* = .90, 95% CI [0.02, 1.687]), and the self-regulation condition had a small effect (*d* = .22, 95% CI [−0.79, 1.07]) on quality-of-life improvement compared with the cancer-fact group
			Race: Asian (Chinese) Gender: all female			
Sherman et al. 2018	Two-arm randomized controlled trial	*N* = 304	Adults with breast cancer stages 1–3 in Australia.	Single online 30-min session. Outcomes assessed at 1 week, 1 month, and 3 months EW: distressing event, body image and self-compassion prompt (MyCB) (*n* = 149) Control: distressing event prompt (EW) (*n* = 155)	Body image distress using 10-item Body Image Scale (BIS), positive body image using 13 item Body Appreciation Scale (BAD), self-compassion using 12-item Self-Compassion Scale – Short Form (SCS-SF)	Participants who received MyCB reported significantly less body image distress (*p* = .035) and greater body appreciation (*p* = .004) and self-compassion (*p* = .001) than control expressive writing participants Moderated by lymphedema status (*p* = .007) and appearance investment (*p* = .042). Self-compassion-mediated effects on both primary outcomes
			Race: Australian ethnicity Gender: all female			
Chu et al. 2019	Three-arm randomized controlled trial	*N* = 96	Adults with breast cancer stage 0–3 in the USA.	30-min sessions once per week over 3 weeks. Outcomes assessed at baseline, 1, 3, and 6 months EW: self-regulation prompt (*n* = 34) Emotional disclosure prompt (*n* = 29) Control: cancer facts prompt (*n* = 33)	PTSD symptoms using PTSD Symptom Scale-Self report (PSS-SR), acculturation using 5-item Stephenson Multigroup Acculturation-Dominant Society Immersion Subscale	Acculturation moderated the effect of expressive writing at 3 and 6 month follow-ups. Among participants with low acculturation, PTSD symptoms were less severe in the self-regulation and cancer-fact groups compared with the emotional disclosure group; in contrast, no group differences in PTSD were found among highly acculturated participants
			Race: Asian (Chinese) Gender: all female			
La Marca et al. 2019	Two-arm randomized controlled trial	*N* = 71	Adults with any cancer type in Italy	Single 15-min session outcomes assessed pre-intervention and at 6 months EW: emotional disclosure prompt (*n* = 35) Control: no EWI (*n* = 36)	General psychopathology using 90-item Symptom Checklist-90-Revised (SCL-90-R), health-related QOL using 36 item Short Form 36-Item Health Survey (SF-36), alexithymia using 20-item Toronto Alexithymia Scale (TAS-20)	The Pennebaker’s EWI was effective in decreasing global psychopathology (*d* = −.55). Small but significant effects were also observed for alexithymia levels and health-related QoL, with the EW showing a reduction in alexithymia levels (*d* = −.31) and an increase in the mental component of QoL (*d* = .31) compared to the Control
			Race: 71 Italian ethnicity Gender: 52.1% males and 47.9% females			
Lu et al. 2019	Three-arm randomized controlled trial	*N* = 90	Adults with breast cancer stages 1–3 in China.	30-min sessions once per week over 4 weeks. Outcomes assessed baseline, 1, and 2 months EW: self-regulation prompt (*n* = 30) Positive thinking prompt (*n* = 30) Control: cancer facts prompt (*n* = 30)	QOL using 37-item Functional Assessment of Cancer Therapy-Breast (FACT-B)	QOL improved overtime in the whole sample. The cancer facts group had increased QOL compared with the self-regulation group from baseline to both the 1 and 2 month follow-ups (ΔQOL = 9.31, *p* = .01, *d* = .44; ΔQOL = 9.45, *p* = .025, *d* = .49). The PTC did not differ from cancer-fact writing but had increased QOL compared with the SRC from baseline to both the 1 and 2 month follow-ups
			Race: Asian (Chinese) Gender: all female			
Ji et al. 2020	Three-arm randomized controlled trial	*N* = 118	Adults with breast cancer stages 0–3 in China.	30-min sessions once per week over 3 weeks. Outcomes assessed at baseline, 3, and 6 months EW: self-regulation prompt (*n* = 31) Emotional disclosure prompt (*n* = 30) Control: cancer facts prompt (*n* = 27)	QOL using 37-item FACT-B	Mainland Chinese breast cancer patients shortly after diagnosis benefit from expressive writing. They benefited more from cancer-facts and emotional disclosure compared to self-regulation. The study indicated that the impact of expressive writing may differ due to stage of cancer survivorship, social, and cultural context
			Race: Asian (Chinese) Gender: all female			
Narayanan et al. 2020	Two-arm randomized controlled trial	*N* = 277	Adults with renal cell carcinoma stages 1–4 in the USA.	Four 20-min sessions over 10 days Outcomes assessed at 1, 4, and 10 months EW: emotional disclosure prompt (*n* = 138) Control: daily facts prompt (*n* = 139)	Religiosity/spirituality using Ironson-Woods Spirituality/Religiousness Index short form, cancer-related symptoms using MDASI, psychological distress using 53-item Brief Symptom Inventory (BSI), fatigue using 9-item BFI, sleep disturbances using 18-item PSQI, social support using 20-item MOS-SSS	Religious Engagement (RE) was associated with reduced cancer-related symptoms over time (*p* = .04), and negative religious coping was associated with increased psychological distress over time (*p* = .004). RE was negatively associated with the BFI scale (*r* = 0.21; *p* < .05) so that the more private RE revealed in one’s writings, the less fatigue was reported. Moreover, negative RC was significantly positively associated with PSQI scores (*r* = 0.23; *p* < .05) so that the more negative RC reported, the greater the sleep disturbances
			Race: 76.1% White Gender: 60% male and 40% female			
Melissant et al. 2021	Two-arm randomized controlled trial	*N* = 233	Adults with head and neck cancer stages 1–4 in the Netherlands.	Single online 30-min session Follow-up 1 week and 1 month EW: distressing event, body image (MyCB) (*n* = 76) Control: no EWI (*n* = 157)	Body image-related distress using 10-item BIS, body appreciation using 10-item Body Appreciation Scale (BAS-2), self-compassion using 12-item Self-Compassion Scale-Short Form (SCS-SF), psychological distress using 14-item Hospital Anxiety and Depression Scale (HADS), health-related QOL using EORTC QLQ-HN43, sexuality using 6-item Female Sexual Function Index (FSFI-6) and 5-item International Index of Erectile Function (IIEF-5)	MyCB does not significantly improve body image-related distress, but is likely to increase self-compassion, which lasts for at least 1 month
			Race: the Netherlands (ethnicity) Gender: 67% male and 33% female			
Mifsud et al. 2021	Three-arm randomized controlled trial	*N* = 79	Adults with breast cancer stages 1–3 in Australia.	Two 30-min sessions per week over 3 weeks Outcomes assessed at baseline, post-intervention, and 1 month EW: distressing event, body image MyCB: self-compassion prompt (*n* = 39) MyCB + meditation (*n* = 17) Control: distressing event prompt (EW) (*n* = 23)	Body image distress using 10-item BIS, body appreciation using 13-item Body Appreciation Scale (BAS), self-compassion using 6-item self-compassionate attitude measure (SCA), trait self-compassion using 12-item Self-Compassion Scale – short form (SCS-SF), positive and negative affect using 20-item Positive and Negative Affect Schedule (PANAS), depression, anxiety, and stress using 21-item short form of the Depression, Anxiety, and Stress Scale (DASS21)	Post-intervention self-compassion and positive affect increased for MyCB compared to EW. At 1-month, Body Image Distress scores decreased across all conditions; self-compassion increased and anxiety decreased for MyCB + M compared to MyCB and EW
			Race: Australian (ethnicity) Gender: all female			
Wu et al. 2021	Two-arm randomized controlled trial	*N* = 112	Adults with breast cancer receiving active treatment in China.	20-min sessions 3 times per week over 4 weeks. Or 20 min over 4 consecutive days. Outcomes assessed at baseline, 1, 3, and 6 months EW: prolonged emotional disclosure prompt (*n* = 56) Control: emotional disclosure prompt (*n* = 56)	QOL using 37-item FACT-B, physical health-related outcomes using 26-item Symptoms Assessment Inventory for Breast Cancer Patients Receiving Chemotherapy (SAI-B), psychological well-being using 14-item HADS	There was no significant difference in the patients’ QOL or physical and psychological well-being between the expressive writing group and the prolonged expressive writing group at any time point (all *p* > .05). The QOL of breast cancer patients significantly decreased in the 2 groups over time
Wang et al. 2022	Two-arm randomized controlled trial	*N* = 82	Adults with breast cancer receiving active chemotherapy in China.	Four 20-min sessions over 4 consecutive days Outcomes assessed baseline, 2, 4, and 6 weeks EW: emotional disclosure, cognitive appraisal, benefit, and future prompt (*n* = 36) Control: no EWI (*n* = 39)	QOL using 39-item QLICP-BR, self-care self-efficacy using 29-item Strategies Used by People to Promote Health scale (SUPPH)	Compared with the control group, the QoL in the intervention group showed a significant and temporary increase at 2 weeks after the intervention (mean difference = −7.56, *p* < .05)
			Race: Asian (Chinese). Gender: all female			
Lu et al. 2023	Three-arm randomized controlled trial	*N* = 136	Adults with breast cancer stages 1–4 in the USA.	EW: self-regulation prompt (*n* = 46) Enhanced self-regulation prompt (*n* = 54) Control: cancer facts prompt (*n* = 36) 30-min sessions once per week over 3 weeks Follow-up baseline, 1, 3, and 6 months	Depressive symptoms using 10-item CES-D, anxiety using 6-item Anxiety subscale of the Brief Symptom Inventory (BSI), perceived stress using 4-item Perceived Stress Scale (PSS), intrusive thoughts using IES	Enhanced self-regulation not the self-regulation prompt, reduced depressive and anxiety symptoms at follow-up time points through reductions in perceived stress
			Race: Asian (Chinese) Gender: all female			
Brkic et al. 2024	Two 2-arm randomized controlled trials	Study 1: *N* = 201; Study 2: *N* = 65	Adults with breast cancer of any stage in Australia.	Study 1: three 15-min sessions for a week (days 1, 4, and 7) Study 2: single 15-min session Outcomes assessed at baseline and 1 week Study 1: Online EYH EW (*n* = 100) Control: creative writing prompt (*n* = 101) Study 2: online EYH EW (*n* = 33) Control: neutral writing/daily facts prompt (*n* = 28)	Body appreciation using short form trait Body Appreciation Scale-2 (BAS-2SF), functionality appreciation using Functionality Appreciation Scale (FAS), body dissatisfaction using 3-items from Body Image States Scale (BISS), state distress using 10-point Likert scale	Study 1 experienced severe attrition; only 14 participants (7 %) completed the intervention and follow-up. Study 2 had higher retention, with 74 % completing the study. In study 2, while no significant differences emerged between EYH and control, both groups significantly improved immediately post-intervention across all outcomes. No differences were found at follow-up
			Race: 93.3% White for study 1; 92.9% White for study 2 Gender: all female			
Nakatani et al. 2024	Two-arm randomized controlled trial	*N* = 15	Adults with Acute Myeloid Leukemia of all stages receiving chemotherapy in the USA.	Four 1-h sessions over 2 weeks Outcomes assessed at baseline, mid-intervention, post-intervention, and 3 months EW: emotional disclosure writing prompt (*n* = 7) Control: neutral writing/daily facts prompt (*n* = 8)	Depression using 8-item Patient Health Questionnaire-8 (PHQ8), anxiety using 7-item Generalized Anxiety Disorder-7 (GAD-7), resilience using 10-item Connor-Davidson Resilience Scale (CD-RISC 10), rumination using 22-item Rumination Response Scale (RRS), QOL using 27-item Functional Assessment of Cancer Therapy-General questionnaire (FACT-G), post-writing reflection using 4-item survey	The intervention demonstrated low feasibility (53% completion; 8/15 participants), with attrition driven by COVID-19 disruptions, high symptom burden, and logistical challenges. While transient improvements emerged post-intervention (e.g., reduced depression/anxiety, enhanced resilience, and short-term emotional/social well-being), no sustained benefits in QOL or mental health outcomes were observed at 3-month follow-up
			Race: 87% White Gender: 53% male and 47% female			
Widanti et al. 2024	Two-arm randomized controlled trial	*N* = 46	Adults with breast cancer post-mastectomy in Indonesia.	Three sessions per week over 1 week Outcomes assessed at baseline, post-intervention, 1 week, 1 month, and 3 months EW: body-image prompt (*n* = 23) Control: no prompt (*n* = 23)	Body image distress using 10-item BIS	Intervention group showed a significant reduction in body image distress (BID) scores (mean reduction: 1.74 ± 1.14, *p* = .000), outperforming the control group (0.35 ± 0.57, *p* = .011), with EW demonstrating a 41.6% effect size (*p* = .000). Improvements in body image distress were sustained at 3-month follow-up; reduced BID correlated with enhanced psychosocial well-being
			Race: not specified Gender: not specified			
Li et al. 2025	Three-arm randomized controlled trial	*N* = 138	Adults with head and neck cancer at any stage receiving radiotherapy in China.	Four 20-min sessions once per week (4 weeks) Outcomes assessed at baseline and post-interventionEW: Benefit-finding (BF) prompt (*n* = 46) Daily facts (NW) (*n* = 47) Control: no writing (*n* = 45)	Psychological distress using 14-item Hospital Anxiety Depression Scale (HADS), nutritional status using Patient-Generated Subjective Global Assessment (PG-SGA), sleep quality using 18-item PSQI	The intervention (EWI) reduced anxiety/depression in the BF and NW groups versus controls (*p* < .05) and improved sleep quality in BF (*p* < .01), though sleep worsened in controls and showed no difference between BF and NW. Nutrition outcomes declined across all groups due to radiotherapy side effects, with no improvements observed
			Race: Asian (Chinese) Gender: 79.7% male and 20.3% female			
Ren et al. 2025	Two-arm randomized controlled trial	*N* = 82	Adults with epithelial ovarian cancer any stage post-surgery receiving chemotherapy in China	Weekly 20-minute session for 6 weeks, starting the second week of chemotherapy Outcomes were assessed at baseline and post-intervention EW: Positive psychology (*n* = 40) Control: no writing (*n* = 42)	Growth level using 21-item Post-Traumatic Growth Inventory (PTGI), anxiety using 20-item Self-Rating Anxiety Scale (SAS), depression using 20-item Self-Rating Depression Scale (SDS), self-evaluation of expressive writing using 5-point Likert scale, expert evaluation of writing content using Kendall’s W consistency test	The experimental group had higher post-traumatic growth (PTGI: 73.43 vs. 63.19, *p* < .001) and greater reductions in anxiety (SAS: 38.25 vs. 45.12) and depression (SDS: 42.10 vs. 48.75) compared to controls
			Race: Asian (Chinese) Gender: all female			
Shin-Cho et al. 2025	Pilot 2-arm randomized controlled trial	*N* = 34	Adults with breast cancer stages 0–4 in the USA	Three sessions lasting 15 min or more over 6 weeks (one every 2 weeks) Outcomes were assessed at baseline and 1-month post-intervention EW: emotional disclosure (*n* = 23) Control: daily facts (*n* = 11)	QOL using 7-item Functional Assessment of Cancer Therapy-General 7 (FACT-G7), perceived stress using 4-item PSS, sleep quality using 19-item PSQI, fatigue using 4-item PROMIS Fatigue Short Form, cancer-related morbidities, depression symptoms using 4-item PROMIS Depression Short Form, anxiety using 4-item PROMIS Anxiety Short Form	The EW demonstrated high feasibility (55% response rate, 86% adherence, 77–78% completion) and acceptability (85% satisfaction, 62% reported stress reduction), with medium effect sizes for improved QOL (*d* = .51) and reduced fatigue (*d* = −.64) in rural breast cancer survivors. While psychosocial outcomes showed promise, broader impacts were not explored
			Race: 74.2% White Gender: all female			
Tan et al., 2025	Two-arm randomized controlled trial	*N* = 76	Adults with breast cancer > 2 months post-treatment in China	Three 20–30-min sessions over 3 weeks Outcomes assessed at baseline and 1-month post-intervention EW: emotional disclosure, narrative expression, and benefit discovery (*n* = 39) Control: no writing (*n* = 37)	QOL using 36-item FACT-B	The intervention group demonstrated significant post-intervention improvements in overall QOL (*p* < .05) and physical well-being (*p* < .05) compared to controls, though no changes were observed in emotional, social/family, or functional well-being
			Race: Asian (Chinese) Gender: all female			
Gripsrud et al. 2016	Qualitative intervention feasibility study	*N* = 7	Adults with breast cancer stages 1–3 post-mastectomy in the USA and Norway	Four sessions at home Essays analyzed at baseline, 3–8 weeks after surgery, and 1–2 weeks after intervention EW: emotional disclosure prompt (*n* = 7)	Semi-structured interviews conducted afterward and analyzed using experiential thematic analysis	Three themes emerged through analysis: writing as a process, writing as therapeutic, and writing as a means to help others. Storytelling encouraged a release of cognitive and emotional strains, surrendering these to reside in the text. The method was said to process feelings and capture experiences tied to a new and overwhelming illness situation, as impressions became expressions through writing
			Race: 71.4% White Gender: all female			
Warmoth et al. 2017	Qualitative intervention study	*N* = 27	Adults with breast cancer stages 1–3 in USA	Three 20–30-min sessions over 3 weeks EW: emotional disclosure prompt (*n* = 27)	The essays were analyzed through 3 phases: first, a phenomenological approach identified meaningful units and synthesized themes/subthemes; second, bilingual researchers independently coded content line-by-line to categorize themes; third, cultural meanings were linked to refine subthemes and build a comprehensive narrative	Emotion suppression, self-stigma, and perceived stigma about being a breast cancer survivor were reflected in the writings. Interpersonally, participants indicated their reluctance to disclose cancer diagnosis to family and friends and concerns about fulfilling multiple roles. Some of them also mentioned barriers of communicating with their husbands Related to life in the USA, participants felt unfamiliar with the healthcare system and encountered language barriers
			Race: Asian (Chinese) Gender: all female			
Przezdziecki et al. 2016	Qualitative intervention acceptability study	*N* = 15	Adults with breast cancer at any stage in Australia	One 30-min session Essays analyzed post-intervention EW: distressing event, body image, and self-compassion prompt (MyCB) (*n* = 15)	Consumers (breast cancer survivors) and health professionals experienced in breast cancer were given access to the website and provided feedback via an online survey regarding their perceptions of the website	Results from both breast cancer survivors and health professionals suggest a moderate to high level of user acceptability and positive ratings for the overall impression of the website
			Race: Australian ethnicity Gender: all female			
Wang et al. 2020	Qualitative intervention study	*N* = 44	Adults with breast cancer stages 0–3 in China	Single 30-min session/week over 3 weeks Essays were analyzed following the intervention EW: self-regulation prompt (*n* = 44)	A phenomenological research method was employed in the data analysis phase that involved collecting and reading the written data, dividing the data into parts, organizing and interpreting the data from a disciplinary perspective, and synthesizing and summarizing the data for communication to the scholarly community	Three themes were identified: treatment decision making, family influences, and cultural influences. Treatment decision-making included the subthemes of preference for mastectomy, passive involvement, and active involvement. Family influences included the subthemes of financial burden, family expectations, and family support. Cultural influences included the subthemes of fatalism, barriers to expressing emotions, and stigma related to cancer
			Race: Asian (Chinese) Gender: all female Location: China			

### Participant characteristics

The 28 included studies examined EWIs in a total of 3527 patients, with individual study sizes ranging from 7 to 507 patients. Twenty studies included only women participants (71.4%). Of the studies conducted in the USA (n = 1089 patients), 46.8% of patients were White, 2.7% were Black, 42.8% were Asian, 3.2% were Latino, and 4.1% identified as Other. Notably, 12 studies only included Asian (Chinese) participants (*n* = 1235), 2 studies only included Australian participants, and 3 studies only included countries of origin (Danish, Indonesian, and Italian) (Table 1). Most studies (71.4%) were conducted on patients with breast cancer, 2 studies on patients with renal cancer (7.1%), 1 study on colorectal cancer (3.6%), 1 study on head and neck cancer (3.6%), 1 study on acute myeloid leukemia (AML), and 1 study on patients with cancer in general without a specific type designated (3.6%).

### Intervention characteristics

Our examined studies described intervention characteristics (e.g., delivery setting, delivery modality) to a varying extent.

### Intervention delivery setting

Intervention delivery settings included patients’ homes (71.4%, *n* = 20), the hospital for cancer treatment (21.4%, *n* = 6), patient preference of home or clinic (3.6%, *n* = 1), or patient preference of home or research office (3.6%, *n* = 1). While most of the interventions were self-administered, some entailed study research assistant phone support in addition to self-administration.

### Intervention delivery modality

Seventy-five percent (*n* = 21) of interventions were delivered via pen-and-paper, 14.3% (*n* = 4) were delivered via an online platform, and 10.7% (*n* = 3) were delivered via pen-and-paper or online based on patient preference. Pen-and-paper formats typically entailed a simple bound journal with blank pages for patients to keep. Online platforms consisted of websites designed by the research team in which patients anonymously typed their journal entries.

### Writing prompts

Most studies (16/28, 57.1%) utilized an emotional disclosure prompt, where participants wrote about their deepest and darkest feelings concerning their cancer and other highly upsetting experiences (Table 1). A similar prompt was utilized by 5 studies (5/28, 17.8%) where they asked participants to write about their body. Four studies utilized the online EWI My Changed Body (MyCB), which is a web-based writing intervention program that uses self-compassion and EW to address adverse body image alterations following cancer treatment (Melissant et al. 2021; Mifsud et al. 2021; Przezdziecki et al. 2016; Sherman et al. 2018). One study used a program called Expand Your Horizon (EYH), which is a writing-based intervention in which participants spend 15 min per session reflecting on and writing about the value of certain body functions (Brkic et al. 2024). For some studies, the control group utilized cancer facts (6/28, 21.4%) or daily facts prompts (8/28, 28.6%), which instructed patients to write about facts relevant to their cancer experience or write about a non-disclosing topic about their daily activities, respectively. A small minority of studies (8/28, 28.6%) utilized a self-regulation or enhanced self-regulation prompt, where the patients either wrote about their deepest feelings at week 1, stress and coping at week 2, and finding benefits at week 3, or wrote about stress and coping at week 1, deepest feelings at week 2, and finding benefits at week 3, respectively. Two studies utilized a positive thinking/psychology prompt where the patients wrote about the positive aspects of their cancer experience (Lu et al. 2019; Ren et al. 2025).

### Timing of intervention

Interventions were delivered after active treatment (19/28, 67.9%), during active treatment (7/28, 25.0%), and at the point of diagnosis (2/28, 7.2%). Of those studies that confined intervention to after active treatment, the time for recruitment ranged from 4 weeks to 5 years. Of the studies that intervened at the point of diagnosis, one study specified within 1 year of diagnosis, while another did not offer a specific time frame other than “newly diagnosed.”

### Timeline of assessments

Studies used different time points for data collection and assessments. While all studies obtained pre-intervention assessments, follow-up assessments were completed at varying time points either proximal to the intervention completion or later such as 10 months post-intervention. Of the 24 quantitative studies, 6 had follow-up assessments at 1, 3, and 6 months, 2 had follow-up at 1, 4, and 10 months, and the rest had variations in follow-up time points (Table 1).

### Study outcome measures

Researchers used a myriad of surveys and scales to assess the impact of their intervention on patients. We broadly grouped them into categories pertaining to psychosocial outcomes, functional outcomes, and medical outcomes (Table 2). Twelve studies assessed health-related QOL (Ji et al. 2020; La Marca et al. 2019; Lepore et al. 2015; Lu et al. 2017, 2018, 2019; Nakatani et al. 2024; Shin-Cho et al. 2025; Sohl et al. 2017; Tan et al. 2025; Wang et al. 2020; Wu et al. 2021), and many other studies focused on other physical symptoms, including sleep (Jensen-Johansen et al. 2018; Lepore et al. 2015; Melissant et al. 2021; Ren et al. 2025; Tan et al. 2025), fatigue (Jensen-Johansen et al. 2018; Melissant et al. 2021; Tan et al. 2025), lymphedema (Milbury et al. 2017), and pain (Jensen-Johansen et al. 2018; Lepore et al. 2015; Melissant et al. 2021). Many of the studies assessed psychosocial outcomes as 9 studies focused on depressive symptoms (Brkic et al. 2024; Jensen-Johansen et al. 2018; Lepore et al. 2015; Lu et al. 2017; Ren et al. 2025; Shin-Cho et al. 2025; Tan et al. 2025; Wang et al. 2022; Widanti et al. 2024), 8 studies looked at anxiety/stress (Brkic et al. 2024; La Marca et al. 2019; Melissant et al. 2021; Nakatani et al. 2024; Ren et al. 2025; Shin-Cho et al. 2025; Tan et al. 2025; Widanti et al. 2024), 4 studies examined self-compassion or self-efficacy (Chu et al. 2019; Lu et al. 2023; Mifsud et al. 2021; Wu et al. 2021), and 5 studies looked at body image distress (Chu et al. 2019; Li et al. 2025; Nakatani et al. 2024; Wang et al. 2022; Wu et al. 2021). Some studies also developed their own tools to assess certain outcomes that did not have pre-existing scales specific to their desired outcome. These assessed the extent of meaningful, personal, and emotional writing participants wrote (Lepore et al. 2015), participants’ state of distress (Brkic et al. 2024), and participant reflections (Widanti et al. 2024).

**Table 2.** Surveys used for outcome measures

Psychosocial	Beck’s Depression Inventory, Visual Analog Scale, Profile of Mood States, Toronto Alexithymia Scale (TAS-20), Marlowe-Crowne Social Desirability and Taylor Manifest Anxiety Scale (for repressive coping style), Emotional Control Questionnaire Rehearsal subscale (for rumination), Profile of Mood States, PTSD Symptom Scale-Self Report (PSS-SR), Self-Compassion Scale-Short Form (SCS-SF), Hospital Anxiety and Depression Scale (HADS = HADS-A + HADS-D), Ironson-Woods Spirituality/Religiousness Index, Brief Symptom Inventory, Medical Outcomes Study Social Support Survey (MOS-SSS), Life Orientation Test-Revised (LOT-R), Impact of Event Scale (IES), Center for Epidemiologic Studies Depression Scale (CES-D), Symptom Checklist-90-Revised (SCL-90-R), Center for Epidemiologic Studies Depression scale (CES-D), Strategies Used By People To Promote Health Scale (SUPPH), Depression, Anxiety and Stress Scale (DASS-21), Generalized Anxiety Disorder-7 (GAD-7), Connor-Davidson Resilience Scale − 10 (CD-RISC 10), Rumination Response Scale (RRS), Post-Traumatic Growth Inventory (PTGI), Self-Rating Anxiety Scale (SAS), Self-Rating Depression Scale (SDS), Perceived Stress Scale (PSS), Positive and Negative Affect Schedule (PANAS), PROMIS Depression Short Form, ROMIS Anxiety Short Form, Social constraints scale (SCS), Perceived Stress Scale
Functional	Functional Assessment of Chronic Illness, Brief Fatigue Inventory, Body Image Scale (BIS), Body Appreciation Scale (BAS-2), Body Appreciation Scale-2 (BAS-2SF), Trait Body Dissatisfaction Scale (MBSRQ-BASS), Female Sexual Function Index (FSFI-6), International Index of Erectile Function (IIEF-5), Brief Fatigue Inventory (BFI), PROMIS Fatigue Short Form, Functional Assessment of Cancer Therapy – General (FACT-G) or breast cancer specific (FACT-BCS), Upper Limb Lymphedema 27 (ULL-27), Pittsburgh Sleep Quality Index (PSQI), Functionality Appreciation Scale (FAS)
Medical	Brief Pain Inventory, Brief Symptom Inventory – Global Severity Index, MD Anderson Symptom Inventory (MDASI), Pittsburgh Sleep Quality Index, Patient Health Questionnaire (PHQ-19), Patient Health Questionnaire-8 (PHQ-8), Number of visits/calls to PCP, Inventory for Breast Cancer Patients Receiving Chemotherapy (SAI-B), Medical Outcomes Study Social Support Survey (MOS-SSS), Short Form 36-Item Survey, Cancer Quality of Life Questionnaire (QLQ-C30), Quality of Life Instruments for Patients with Breast Cancer (QLICP-BR), Subjective Global Assessment (PG-SGA), European Organization for Research and Treatment of Cancer Head and Neck Cancer Module (EORTC QLQ-HN43)

### Benefits of EWIs

#### Quantitative studies

Of the 24 quantitative studies, 87.5% (*n* = 21) highlighted potential benefits such as improved quality of life (QOL) and health-related symptoms (Table 1). Major outcomes positively impacted by EWI in patients with cancer from the studies included QOL, global well-being, physical symptoms, including sleep and fatigue, body image, and self-efficacy/compassion.

#### Quality of life

Twelve studies focused on the impact of EWIs on QOL. Of the 12 studies that assessed the impact of an EWI on QOL, 8 showed a positive significant association between EWIs and QOL (Ji et al. 2020; La Marca et al. 2019; Lu et al. 2017, 2018, 2019; Shin-Cho et al. 2025; Tan et al. 2025; Wang et al. 2022), and 4 showed a negative or no association between EWIs and QOL (Lepore et al. 2015; Nakatani et al. 2024; Sohl et al. 2017; Wu et al. 2021). An example of a study highlighting a positive association between EWIs and QOL was done by LaMarca and colleagues who used a 2-armed RCT in 71 patients randomized to an emotional disclosure EWI or cancer facts control group to show that a Pennebaker-modeled EWI improved health-related QOL for patients with cancer when compared to control (*d* = .31) (La Marca et al. 2019). Wang and colleagues conducted a 2-arm RCT with 82 patients with breast cancer randomized to an emotional disclosure EWI or control group and showed that EWI significantly improved QOL at 2 weeks when compared to the control group (*p* < .05) (Wang et al. 2022). Lastly, Tan and colleagues conducted a 2-arm RCT with 76 patients with breast cancer randomized to an emotional disclosure EWI and routine care control group and found that compared to the control group, the EWI group exhibited significant improvements in QOL (*p* <.05) (Tan et al. 2025).

An example of a study that found a negative association between EWI and QOL was done by Wu and colleagues, who conducted a 2-arm RCT with 112 patients with breast cancer, showing that QOL significantly decreased over time for both the experimental and control groups (*p* < .05), and there was no significant difference in QOL between the 2 groups (*p* > .05) (Wu et al. 2021). Interestingly, Sohl and colleagues conducted a 2-armed RCT with 104 patients with breast cancer randomized to an emotional disclosure EWI or daily facts control group and found that EWIs did not provide statistically significant main or interaction impacts on any QOL measure (*p* > .05). However, EWIs were more effective for improving QOL in women who were higher on scales of dispositional optimism (LOT-R) (*p* = .017), lower on scales of avoidant behavior (IES-avoidance) (*p* = .007), and had less time since lymphedema diagnosis (*p* = .003) (Sohl et al. 2017).

#### Well-being globally

Eight studies found that EWI led to significantly improved global well-being (e.g., depression (Brkic et al. 2024; Nakatani et al. 2024; Ren et al. 2025), anxiety, and perceived stress (La Marca et al. 2019; Li et al. 2025; Lu et al. 2023; Nakatani et al. 2024; Ren et al. 2025), emotional/social/physical well-being (Lu et al. 2017; Tan et al. 2025), PTSD symptoms (Chu et al. 2019; Ren et al. 2025), and resilience (Nakatani et al. 2024)). For example, Ren and colleagues conducted a 2-arm RCT with 82 patients with epithelial ovarian cancer randomized to a positive psychology EWI or a routine care control group and showed that compared to the control group, the experimental group had greater reductions in depression (mean ± SD: 42.10 ± 3.86 vs. 48.75 ± 4.62, *p* < .001) (Ren et al. 2025). Li and colleagues conducted a 3-arm RCT with 138 patients with head and neck cancer receiving radiotherapy randomized to a benefit finding EWI, daily facts EWI, or routine care control group and found that both writing groups reduced depression and anxiety when compared to controls (*p* < .05). Tan and colleagues also observed significant improvements in physical well-being for their EW group when compared to control, in addition to the improvements in QOL mentioned above (Tan et al. 2025).

PTSD symptoms were also improved with EWIs in 2 studies. Chu and colleagues conducted a 3-arm RCT with 96 patients with breast cancer randomized to a self-regulation EWI, emotional disclosure EWI, or cancer facts control group and found that among patients with low acculturation, PTSD symptoms were less severe in the self-regulation and cancer-fact groups when compared to the emotional disclosure EWI (*p* < .05) (Chu et al. 2019). In Ren and colleagues’ two-arm RCT with 82 patients with epithelial ovarian cancer, they also found that the positive psychology EWI had significantly higher post-traumatic growth (PTGI: 73.43 vs. 63.19, *p* < 0.001) when compared to routine care controls (Ren et al. 2025).

Nakatani showed that EW significantly enhanced resilience in their 2-arm randomized pilot study with 46 patients with AML randomized to an emotional disclosure EWI and daily facts writing control, where transient yet significant improvements in resilience were present for the EW group compared to controls (no *p* values provided) (Nakatani et al. 2024).

#### Physical symptoms including sleep and fatigue

Five studies highlighted EWI’s positive impact on physical symptoms (Jensen-Johansen et al. 2018; Milbury et al. 2017), sleep (Li et al. 2025; Milbury et al. 2017; Narayanan et al. 2020), and fatigue (Shin-Cho et al. 2025). Milbury and colleagues conducted a 2-arm RCT with 277 patients with renal cell carcinoma randomized to an emotional disclosure EWI or a daily fact-writing control group and found that compared to controls, the EWI group had significant improvements in cancer-related symptoms (*p* < .05) and sleep disturbances (*p* < .005) (Milbury et al. 2017). Jensen-Johansen and colleagues conducted a 2-arm RCT with 507 patients with breast cancer randomized to an emotional disclosure EWI or daily facts-writing control group and found that low-alexithymia women in the EW group showed larger decreases in general practitioner telephone calls over time than both high-alexithymia women and controls (*p* = .006) (Jensen-Johansen et al. 2018). In Shin-Cho and colleagues’ 2-arm RCT, they showed that the EWI group had significantly reduced fatigue (*d* = −.64) when compared to the daily facts control group (Shin-Cho et al. 2025). Li and colleagues’ 3-arm RCT also showed that when compared to routine care controls, the benefit-finding EWI group showed improved sleep quality (*p* < .01) (Li et al. 2025). Interestingly, Narayanan and colleagues conducted a novel 2-arm RCT, examining the religious engagement in EW for 277 patients with renal cell carcinoma randomized into an emotional disclosure EWI or a daily facts control group, and found that religious engagement was negatively associated with fatigue (*r* = 0.21; *p* < 0.05), and negative religious content (less religious content) was significantly positively associated with poor sleep (*r* = 0.23; *p* < 0.05) (Narayanan et al. 2020).

#### Body image, self-efficacy/compassion

Four studies found that EWI’s positively impacted body image perception (Sherman et al. 2018; Widanti et al. 2024) and self-efficacy/compassion (Melissant et al. 2021; Mifsud et al. 2021; Sherman et al. 2018). For example, Widanti and colleagues conducted a 2-arm RCT with 46 patients with breast cancer randomized to a body image EWI and free writing control group and found that the body image EWI group showed a significant reduction in body image distress (mean reduction: 1.74 ± 1.14, *p* = .000), compared to the control group (0.35 ± 0.57, *p* = .011), with EW demonstrating a 41.6% effect size (*p* = .000) with sustainment of these improvements at 3-month follow-up (Widanti et al. 2024). Sherman and colleagues conducted a 2-arm RCT with randomization of 304 patients with breast cancer into a MyCB EWI or distressing event EWI control group and found that participants who received MyCB reported significantly less body image distress (*p* = .035), greater body appreciation (*p* = .004), and self-compassion (*p* = .001) than the control group (Sherman et al. 2018). Melissant and colleagues conducted a 2-arm RCT with 233 patients randomized to a MyCB EWI or routine care control group and found that the MyCB group showed significant improvement in self-compassion (*p* = .003) (Melissant et al. 2021). Additionally, Mifsud and colleagues ran a 3-arm RCT with 79 patients with breast cancer randomized to a MyCB EWI, MyCB + meditation, or distressing event EWI control group and found that self-compassion and positive affect increased for MyCB compared to the EWI control group (*p* = .002 and .046, respectively), and at 1-month, body image distress decreased across all conditions (*p* = .02); self-compassion increased and anxiety decreased for MyCB + M compared to MyCB EWI and the EWI control group (*p* = .002 and .001, respectively) (Mifsud et al. 2021).

#### Modifiers of EWI benefits

Additionally, 11 studies identified modifier effects on outcomes as follows: the cancer fact, self-regulation, positive thinking, and self-compassion EW prompts, high optimism, low avoidance, early cancer survivorship, having social support and more severe depressive symptoms, low alexithymia, lymphedema status, self-compassion, low acculturation, social and cultural contexts, religious engagement and coping, and meditation (Chu et al. 2019; Jensen-Johansen et al. 2018; Ji et al. 2020; Lu et al. 2019, 2018, 2017; Mifsud et al. 2021; Milbury et al. 2017; Narayanan et al. 2020; Sherman et al. 2018; Sohl et al. 2017). Three studies examined potential additional modifiers and found them to be noncontributory, including sex, social constraints, repressive coping, rumination, writing topic, and writing dosage (Jensen-Johansen et al. 2018; Lepore et al. 2015; Wu et al. 2021) (Table 3).

**Table 3.** Modifiers of EWI impact on quality of life (QOL) or health-related outcomes

Positive effect modifiers	Studies identifying	Outcome
Cancer facts prompt	Ji et al. 2020; Lu et al. 2017, Lu et al. 2019; Li et al., 2025; Shin-Cho et al. 2025	QOL; depression, anxiety, and sleep
Emotional disclosure prompt	Ji et al. 2020; Nakatani et al. 2024; Tan et al., 2025; Shin-Cho et al. 2025	QOL
Positive thinking prompt	Lu et al. 2019; Ren et al.; 2025	QOL; post-traumatic growth, depression, and anxiety
Order of self-regulation prompt	Lu et al. 2018	QOL
High optimism	Sohl et al. 2017	QOL
Low avoidance	Sohl et al. 2017	QOL
Early cancer survivorship or lymphedema status	Ji et al. 2020; Sherman et al. 2018; Sohl et al. 2017	QOL
Social support + elevated depressive symptoms	Milbury et al. 2017	Reduced cancer-related, depressive, and sleep symptoms
Low alexithymia	Jensen-Johansen et al. 2018	Reduced physical symptoms and healthcare utilization
MyCB self-compassion prompt	Melissant et al. 2021; Mifsud et al. 2021; Sherman et al. 2018	Low body image distress, self-compassion, body appreciation, and appearance investment
Low acculturation	Chu et al. 2019	Reduced PTSD symptoms
Social and cultural context	Ji et al. 2020	QOL
Religious engagement and coping	Narayanan et al. 2020	Reduced cancer-related symptoms
Meditation	Mifsud et al. 2021	Self-compassion decreased anxiety

### Qualitative studies

Four qualitative studies were included in our review, with a total of 93 patients. Of these studies, 1 was conducted in the USA and Norway (Gripsrud et al. 2016), 1 was conducted in the USA (Warmoth et al. 2017), 1 was conducted in Australia (Przezdziecki et al. 2016), and 1 was conducted in China (Wang et al. 2020). Three of the studies analyzed the patients’ writings (Gripsrud et al. 2016; Wang et al. 2020; Warmoth et al. 2017), and 1 study examined the feasibility of providing an EWI through an online format (MyCB) (Przezdziecki et al. 2016). The insights from these qualitative studies highlight mechanisms by which EWIs support patients with cancer. Three themes emerged from the synthesis of these studies: (1) *EWIs facilitate the narrative reconstruction of cancer-related trauma*, by enabling the processing of existential distress through the act of storytelling; (2) *disclosure is shaped by cultural norms*, where the psychosocial context of patients’ environments shape what they disclose through their writings; and (3) *the delivery format of interventions matters*, as online platforms like MyCB help dismantle barriers to emotional expression in cultural environments that prioritize emotional restraint. Overall, these qualitative studies support that the therapeutic value of EWIs extends beyond emotional release by validating patients’ agency, such as reasserting control over treatment decisions (Wang et al. 2020) or reclaiming bodily autonomy post-mastectomy (Gripsrud et al. 2016). These findings uncover the importance of adapting EWIs cross-culturally (Wang et al. 2020; Warmoth et al. 2017) and tailoring prompts (Gripsrud et al. 2016) and mechanisms of delivery (Gripsrud et al. 2016; Przezdziecki et al. 2016) to help navigate the sociocultural barriers that prevent patient disclosure.

#### Narrative reconstruction

EWIs enable patients with cancer to reconstruct overwhelming traumatic cancer experiences into coherent narratives through the process of storytelling. This exercise helps to foster agency, psychological resilience, and reshape the meaning of the experiences that patients with cancer go through. For example, Gripsrud and colleagues conducted a qualitative intervention feasibility study of an emotional disclosure EWI with 7 post-mastectomy patients with breast cancer in the USA and Norway and found that breast cancer survivors utilized writing through the EWI to convert “impressions into expressions,” restructuring situational distress into survivorship-affirming stories (Gripsrud et al. 2016). One patient expressed that the EWI enabled her to let her thoughts “reside somewhere else,” helping her to develop agency in reducing her cognitive load. Additionally, Wang and colleagues conducted a qualitative intervention study with 44 patients with breast cancer in mainland China and found that their self-regulation EWI helped survivors to process existential distress, such as the reconciliation of mastectomy decisions in the cultural contexts of femininity (Wang et al. 2020). One patient wrote, “As long as it would save my life, any sacrifice was worth it,” highlighting reconstruction of loss or sacrifice into agency. EWIs are not solely emotional vectors for patients with cancer, but tools that help reconstruct cancer-related traumas and provide survivorship-affirmation.

#### Cultural disclosure norms

Cultural norms and values often inform how and to whom patients disclose emotions and experiences. As such, EWIs must be culturally adaptive to enable safe disclosure for patients with cancer. For example, Warmoth and colleagues conducted a qualitative emotional disclosure EWI study with 27 Chinese immigrant patients with breast cancer in the USA and found that the EWI provided an avenue of disclosure that Confucian and collectivist cultural values may inhibit (Warmoth et al. 2017). One patient wrote, “I didn’t even tell my husband and daughter. I was afraid they would be distressed. So, I was the only one who knew this (breast cancer diagnosis).” This patient feared being perceived as a burden, a common concern fueled by cultural stigma around cancer as a “punishment” or “bad luck”(Warmoth et al. 2017). Similarly, in Wang and colleagues’ study with 44 Chinese mainland breast cancer survivors further supports these cultural barriers, as one patient wrote that “I didn’t tell my mother about the diagnosis because I was worried that she could not accept the truth. I didn’t tell my son about the diagnosis because I didn’t want him to worry about it. I didn’t tell my husband about the diagnosis because I didn’t want to burden him. Sometimes, I felt like I was wearing a mask to live” (Wang et al. 2020). This cultural barrier contrasts with patients in Western cultures, where participants openly discussed grief and body image struggles without familial cultural restraints. For example, in Gripsrud and colleagues’ study with breast cancer survivors in the USA and Norway, one breast cancer survivor said, “I think I’m probably going to let my family read it, maybe put… what I’ve written into a little book or diary, and have them read it,” suggesting that familial cultural norms are not as apparent in this Western environment. This stark contrast highlights how cultural norms can impact disclosure in patients with cancer and underscores the importance of implementing EWIs that respect cultural differences (e.g., framing prompts around collectivist familial roles rather than individualism or conducting the EWI in patients’ native language) (Wang et al. 2020).

#### Intervention delivery format

Home-based EWIs, especially on online platforms, help dismantle cultural barriers to emotional disclosure, particularly in contexts where face-to-face disclosure may be stigmatized. In Przezdziecki and colleagues’ qualitative self-regulation EWI acceptability study with 15 patients with breast cancer in Australia, they utilized the web-based EWI “MyCB,” and patients appreciated the privacy it provided for their disclosure (Przezdziecki et al. 2016). One patient noted, “I could express things on paper I wouldn’t tell my family,” highlighting how the digital platform provided evasion from cultural punishment against public vulnerability. Additionally, health professionals reviewing the web-based writings noted its ability to circumvent potential stigma, as patients could anonymously write without fear of judgment, a common cultural barrier impacting emotional disclosure. Similarly, Wang and colleagues found that the mainland Chinese breast cancer survivors in their study preferred home-based writing and structured prompts, as it helped to avoid public emotional displays and to express their fears safely (Wang et al. 2020). In sum, EWIs may be culturally adapted to enhance their therapeutic effect and promote emotional disclosure for patients from all cultural backgrounds.

## Discussion

This scoping review synthesized findings from EWIs in 28 studies of 3527 patients with cancer, with quantitative and qualitative data as well as potential associations between EWIs and outcomes. Twenty-four studies examined and reported the association between EWIs and clinical outcomes (e.g., symptoms, psychological distress, and QOL) using validated instruments and quantitative assessments pre-post intervention, while 4 studies used qualitative methods to provide insights into patient experiences with EWIs (Tables 1 and 2). While most studies suggest potential benefits of EWIs for patients with cancer, further longitudinal research is needed to establish the overall impact of EWIs in these populations.

Although quantitative and qualitative studies highlight the potential benefit of EWIs for patients with cancer, the heterogeneity of these studies makes it difficult to translate these findings into clinical practice. For example, studies assessed the same outcomes with different validated instruments such as QOL being assessed with the Cancer Quality of Life Questionnaire (QLQ-C30) (Aaronson et al. 1993) in 1 study (Lepore et al. 2015), Functional Assessment of Cancer Therapy – Breast scale (Wan et al. 2007) (FACT-B) (Ji et al. 2020; Lu et al. 2019; Sohl et al. 2017; Tan et al. 2025; Wu et al. 2021) or Functional Assessment of Cancer Therapy general scale (FACT-G) (Cella and Tulsky 1993) in 8 studies (Lu et al. 2018, 2017; Nakatani et al. 2024), QOL Instruments for Patients with Breast Cancer (QLICP-BR) (Wan et al. 2009) in 1 study (Wang et al. 2022), and European Organization for Research and Treatment of Cancer Head and Neck Cancer Module (EORTC QLQ-HN43) (Aaronson et al. 1993) in 1 study (Melissant et al. 2021). Additionally, the method of conducting EWIs varies significantly between the studies in this review, as differences in the timing of intervention with ongoing treatment for patients, timeline of assessing outcomes, intervention modality and settings were present. Heterogeneity in the methodology of RCT’s assessing the benefits of EWIs in patients with cancers underscored the conclusions presented by the 2 previous reviews conducted on EWIs and their impacts on patients with cancer (Merz et al. 2014; Zachariae and O’Toole 2015). Additionally, some potential benefits of EWI’s including their ability to provide safe emotional disclosure, as supported by the qualitative data in this study (Gripsrud et al. 2016; Przezdziecki et al. 2016; Wang et al. 2020; Warmoth et al. 2017), are not easily captured by validated measures, highlighting the importance of qualitative studies within this topic.

The demographics of patient populations in recent studies are limited, and future interventions must foster more inclusivity. Cancer-risk behaviors are more prevalent in men than women, and men are more likely to die from cancer (Cook et al. 2011; Pinkhasov et al. 2010). Despite these gender differences, the studies in this scoping review rarely included men or the cancers that predominantly impact men. Sixty-six percent of the studies analyzed, encompassing 16 of the 24 quantitative studies, and all 4 of the qualitative studies focused on breast cancer and only included women. Traditional male gender norms inhibit the emotional expression in men with cancer (Hoyt 2009), and Smyth’s meta-analysis theorized that men would benefit more from EWI’s than women due to societal norms that restrict emotional expression in men (Smyth 1998). Accordingly, EWIs could serve as a valuable outlet to promote emotional disclosure for men with cancer. Incorporating gender considerations into the design of health programs and studies strengthens the literature and improves patient outcomes (Griffith et al. 2011), as such investigators and clinicians exploring EWIs for patients with cancer should be mindful of the influence of gender on biopsychosocial factors that may impact these interventions.

Additionally, there is a significant gap in racial and ethnic representation in the literature on EWIs and cancer, particularly concerning non-White or non-Asian patients. Despite most studies focusing on cancers in the USA, the represented patient populations do not reflect the diversity seen in clinical practice (Turner et al. 2022). Reassuringly, the current literature provides that when racial- and ethnic-specific studies are conducted, they yield insightful results. From the 12 studies that only included patients of Asian (Chinese) descent, insights regarding the impact of acculturation, language, Confucian and collectivist culture, stigma, and social constraints were provided. Utilizing these studies as models for research in historically underrepresented racial and ethnic groups may offer critical insights into best practices for implementing EWIs as adjunctive therapy in cancer treatment.

The synthesis of this review provides insights for investigators and clinicians considering integrating EWIs into cancer care. First, EWIs should be tailored to the cultural and individual needs of patients with cancer. For example, the qualitative data synthesized in this study suggest that patients who come from cultures with more collectivist values may resonate with the privacy and safety that EWIs provide (Przezdziecki et al. 2016; Wang et al. 2020) and benefit from prompts structured around their cultural values and in their native language (Wang et al. 2020). Second, EWIs should prioritize delivery formats that prioritize privacy and accessibility, such as online formats (e.g., MyCB and EYH) (Brkic et al. 2024; Melissant et al. 2021; Mifsud et al. 2021; Przezdziecki et al. 2016; Sherman et al. 2018), especially for sensitive topics such as body image distress. Third, interventions should be timed strategically to prioritize emotional disclosure and maximize therapeutic benefits. Most studies indicating a positive impact on clinical outcomes for EWIs in patients with cancer involved interventions delivered after active treatment when patients may have had a greater capacity for reflection and engagement with the EWI. Fourth, future research must address inclusivity gaps by expanding EWI investigations to cancer predominantly affecting men, and clinicians should encourage their patients with cancer who are men to engage in emotional disclosure exercises, such as EWIs, given theoretical evidence suggesting its benefit for this population (Hoyt 2009; Smyth 1998). Fifth, investigators must standardize outcome measures in studies assessing EWI’s impact on this patient population, to decrease heterogeneity. Although our synthesis identifies positive associations between EWIs and clinical outcomes, the heterogeneity present in recent studies limits the conclusions that can be made.

There were several limitations in this study that should be noted. First, the reviewed studies were impacted by patient-reported outcome biases, and heterogeneity in the protocol for EWIs, writing prompts, follow-up time, and outcome measures, highlighting the continued need for rigorous studies designed to maximize patient inclusivity, generalizability, and outcomes. Furthermore, although rigorous efforts were made to conduct the search strategy for the current scoping review, this study may not include all available literature on EWIs in patients with cancer. Lastly, by design, this study does not include meticulous meta-analysis or appraisal of the data presented by the studies in the review. As such, future studies may uncover insights and findings from the data in the studies of this review that were not elucidated by our study design. However, the purpose of this scoping review was to present a synthesis of the existing and developing body of literature regarding EWIs in patients with cancer and to identify gaps that may provide areas of improvement for future studies.

## Conclusion and future recommendations

This scoping review synthesizes the quantitative and qualitative data from 28 studies (2015–2025) on EWIs’ potential benefits in cancer care. Our synthesis provides that EWIs are predominantly positively associated with clinical outcomes in patients with cancer, and qualitative insights provide that EWIs offer safe emotional disclosure through narrative reconstruction and culturally adaptive delivery. Studies lack gender, racial, and ethnic diversity, highlighting the need for investigations of EWIs in men and underrepresented minority patients (e.g., Black and Latino) with cancer. Culturally tailored interventions and privacy-focused online delivery demonstrate promise in addressing perceived and experienced stigma in this patient population. Timing EWIs post-treatment may optimize benefits, and future research must standardize EWI protocols to strengthen conclusions on the efficacy of integrating EWIs into cancer care.

## Supporting information

10.1017/S1478951525100394.sm001 Watson et al. supplementary material Watson et al. supplementary material

## Supplementary material

The supplementary material for this article can be found at https://doi.org/10.1017/S1478951525100394.
