# Supplementary material for: Expressive writing interventions in patients with cancer: A scoping literature review
Source: Palliat Support Care. 2025 Jul 21;23:e132. doi: 10.1017/S1478951525100394 (PMC13166703; doi:10.1017/S1478951525100394)
Supplement: Watson et al. supplementary material [file S1478951525100394sup001.docx]

**Supplementary Tables**

**Supplementary Table 1. Non-Modifiers of EWI impact on QoL or Health Related Outcomes**

| **Non-Modifiers** | **Studies Identifying** | **Outcome** |
| --- | --- | --- |
| Sex | (Lepore, Revenson, Roberts, Pranikoff, & Davey, 2015a) | **QoL** |
| Social Constraints | (Jensen-Johansen et al., 2018; Lepore, Revenson, Roberts, Pranikoff, & Davey, 2015a) | QoL, Physical Symptoms and Healthcare Utilization |
| Repressive Coping | (Jensen-Johansen et al., 2018) | Physical Symptoms and Healthcare Utilization |
| Rumination | (Jensen-Johansen et al., 2018) | Physical Symptoms and Healthcare Utilization |
| Writing Prompt | (Jensen-Johansen et al., 2018) | Physical Symptoms and Healthcare Utilization |
| Writing Dosage | (Wu et al., 2021) | QoL, Physical or Psychological well-being |
